# Small bowel leiomyosarcoma: a case report and review of the literature

**DOI:** 10.1093/jscr/rjaf269

**Published:** 2025-05-03

**Authors:** Rebaz O Mohammed, Rawa M Ali, Deari A Ismaeil, Rebaz M Ali, Hemn H Kaka Ali, Karokh F Hamahussein, Omar H G Hawramy, Hiwa O Abdullah, Marwan A Ghafoor, Fahmi H Kakamad

**Affiliations:** Scientific Affairs Department, Smart Health Tower, Madam Mitterrand Street, Sulaymaniyah 46001, Iraq; Scientific Affairs Department, Smart Health Tower, Madam Mitterrand Street, Sulaymaniyah 46001, Iraq; Hospital for Treatment of Victims of Chemical Weapons, Mawlawy Street, Halabja 46018, Iraq; Scientific Affairs Department, Smart Health Tower, Madam Mitterrand Street, Sulaymaniyah 46001, Iraq; College of Medicine, University of Sulaimani, Madam Mitterrand Street, Sulaymaniyah 46001, Iraq; Scientific Affairs Department, Smart Health Tower, Madam Mitterrand Street, Sulaymaniyah 46001, Iraq; Oncology Department, Hiwa Hospital, Shorsh Street, Sulaymaniyah, Iraq; Scientific Affairs Department, Smart Health Tower, Madam Mitterrand Street, Sulaymaniyah 46001, Iraq; Gastroenterology and Hepatology Teaching Hospital, Zanko Street, Sulaymaniyah 46001, Iraq; Scientific Affairs Department, Smart Health Tower, Madam Mitterrand Street, Sulaymaniyah 46001, Iraq; Gastroenterology and Hepatology Teaching Hospital, Zanko Street, Sulaymaniyah 46001, Iraq; Scientific Affairs Department, Smart Health Tower, Madam Mitterrand Street, Sulaymaniyah 46001, Iraq; Gastroenterology and Hepatology Teaching Hospital, Zanko Street, Sulaymaniyah 46001, Iraq; Scientific Affairs Department, Smart Health Tower, Madam Mitterrand Street, Sulaymaniyah 46001, Iraq; Kscien Organization for Scientific Research (Middle East office), Hamdi Street, Azadi Mall, Sulaymaniyah 46001, Iraq; School of Health Sciences, Health Campus, Universiti Sains Malaysia, Kubang Kerian, 15200 Kota Bharu, Kelantan, Malaysia; Scientific Affairs Department, Smart Health Tower, Madam Mitterrand Street, Sulaymaniyah 46001, Iraq; College of Medicine, University of Sulaimani, Madam Mitterrand Street, Sulaymaniyah 46001, Iraq; Kscien Organization for Scientific Research (Middle East office), Hamdi Street, Azadi Mall, Sulaymaniyah 46001, Iraq

**Keywords:** small intestine, leiomyosarcoma, abdominal tumor, sarcoma

## Abstract

Leiomyosarcoma of the small intestine is a rare malignancy. Despite advancements in diagnostic imaging, leiomyosarcoma remains challenging to diagnose preoperatively due to its nonspecific presentation. This study presents a 72-year-old female with a 3 cm ileal leiomyosarcoma, diagnosed via histopathology. Post-surgery, she recovered well with no recurrence at 4 months. A review of 13 cases showed ages 45–90 years (mean: 69.8), with males affected twice as often. Abdominal pain was the most common symptom, and metastases occurred in several cases to the lungs, liver, and other organs. The mortality rate was 38.5%. Early diagnosis is crucial, as mild symptoms may be overlooked until severe complications arise.

## Introduction

Malignant small bowel tumors account for <5% of gastrointestinal malignancies, with sarcomas comprising 1.2% [1, 2]. Common types include carcinoids, adenocarcinomas, lymphomas, gastrointestinal stromal tumors (GISTs), and leiomyosarcoma (LMS). LMS, a rare tumor, is most frequent in the jejunum (32%), ileum (25.2%), and duodenum (12.6%) [3]. Patients often present with nonspecific symptoms such as pain or obstruction, complicating diagnosis [4]. Peak incidence occurs in males in their 60s, with nonsteroidal anti-inflammatory drug use possibly contributing [5]. Herein, a case of LMS is reported in an elderly female patient who presented with paroxysmal abdominal pain. The eligibility of the references has been confirmed, and the case has been written according to the CaReL guidelines [6, 7].

## Case presentation

### Patient information

A 72-year-old female presented with chronic paroxysmal abdominal pain, progressively worsening over the past 5 months. Her past medical history included hypertension, diabetes mellitus, and hyperthyroidism, with previous surgical interventions including paraumbilical hernia repair surgery and open abdominal surgery for an unspecified bowel-related illness, with no records.

### Clinical findings

On physical examination, her abdomen appeared distended, with a large, ~20 × 25 cm swelling in the paraumbilical region, where a previous surgical scar was visible. The swelling was tense and irreducible, with mild tenderness but without skin discoloration or other significant features. Bowel sounds were normal.

### Diagnostic assessment

Laboratory tests showed hyperglycemia (glucose: 345 mg/dl, HbA1c: 8.9%), indicating poorly controlled diabetes. Kidney function was normal (creatinine: 0.57 mg/dl, urea: 20.5 mg/dl). Complete blood count (CBC) revealed leukocytosis (WBC: 11.8 × 10^9^/l), anemia (RBC: 3.03 × 10^12^/l, Hb: 8.3 g/dl, Hct: 24.8%), and normal platelets (290 × 10^9^/l).

Ultrasound detected a 35 × 24 mm anterior abdominal wall hernia with a bowel loop. Esophagogastroduodenoscopy showed gastric erosions but no ulcers or masses. Histopathology confirmed chronic active gastritis with mild atrophy and rare *Helicobacter pylori* like structures.

Colonoscopy, limited to the splenic flexure, revealed normal mucosa and six small sessile polyps, removed via biopsy forceps and cold snare. Histopathology identified tubular adenomas with low-grade dysplasia and goblet cell-type hyperplastic polyps, without high-grade dysplasia or carcinoma.

### Therapeutic interventions

The patient underwent surgical resection. A transverse elliptical incision included excision of the old scar. Dissection exposed the hernial sac, containing the sigmoid colon and greater omentum, without ischemia. A 3 × 3 cm mass on the mesenteric border of the small bowel was resected along with 15 cm of normal bowel for adequate margins. The defect was closed without tension or mesh, and a Redivac drain was placed.

**Figure 1:**
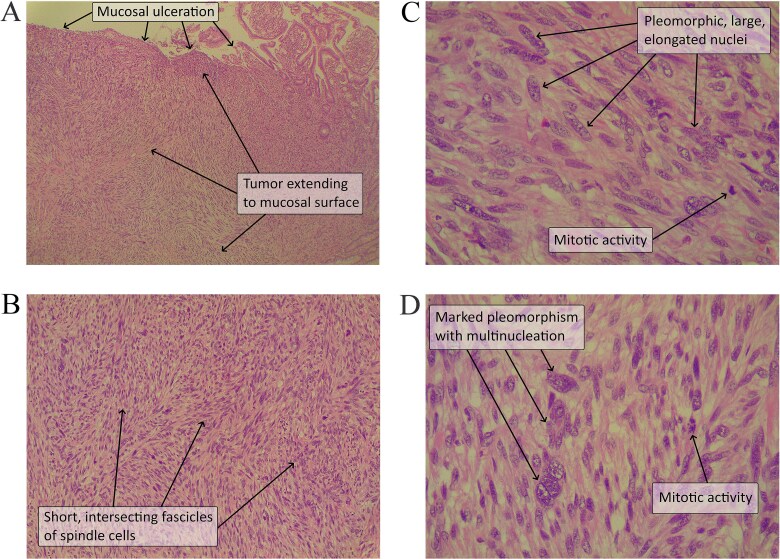
(A) The tumor extends from the ulcerated mucosa down to the deeper layers. (B) The spindled tumor cells are arranged as short intersecting fascicles. (C–D) The tumor cells have large, pleomorphic, elongated nuclei with vesicular chromatin and irregular nuclear outlines. Mitotic activity is easily identifiable [hematoxylin and eosin; original magnification × 40 (A), × 100 (B), × 400 (C and D)].

Histopathology revealed a 3-cm polypoid tan-white mass occupying the lumen, with spindle cells arranged in fascicles, pleomorphic nuclei, and high mitotic activity (19/1 mm^2^). No necrosis was observed, classifying it as high-grade sarcoma (FNCLCC score 6, pT1) (Fig. 1).

**Figure 2:**
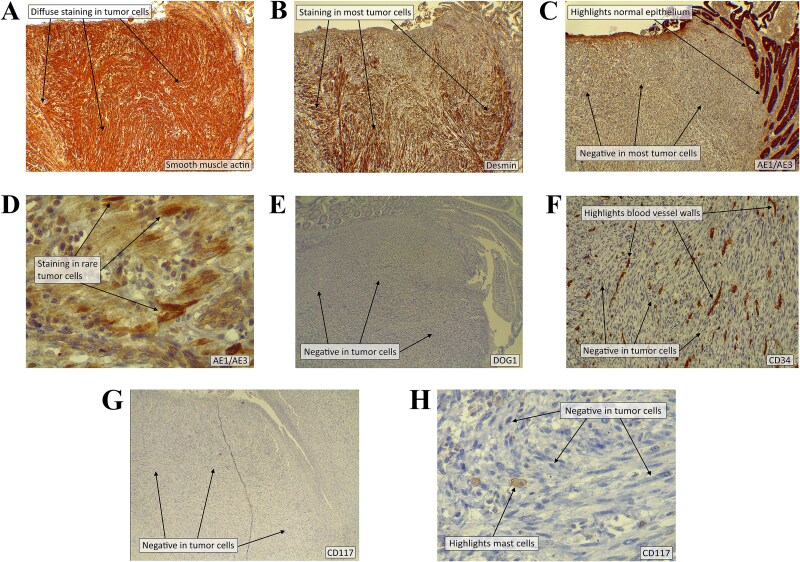
(A) There is cytoplasmic staining for SMA of strong intensity in 100% of the tumor cells. (B) There is cytoplasmic staining for DS of strong intensity in 90% of the tumor cells. (C) The majority of the tumor cells are negative for the pan-keratin marker AE1/AE3. The stain highlights the normal surface epithelium and crypts. (D) A few tumor cells (5%) show cytoplasmic staining of moderate intensity for AE1/AE3. (E) The tumor cells are negative for DOG1. (F) The tumor cells are negative for CD34. The stain highlights the wall of normal blood vessels. (G–H) The tumor cells are completely negative for CD117 (using two different clones). The stain highlights normal mast cells. [IHC for various antibodies using diaminobenzidine chromogen; original magnification × 40 (A–C, E, and G), × 100 (F), × 400 (D and H)].

Immunohistochemistry (IHC) showed strong positivity for smooth muscle actin (SMA) (100%) and desmin (DS) (90%), weak AE1/AE3 reactivity (5%), and negativity for CD117, DOG1, and CD34 (Fig. 2). These findings confirmed LMS, excluding GISTs and sarcomatoid carcinoma.

### Follow-up

The postoperative period was uneventful, and the patient resumed her daily activities. After 4 months of follow-up, her bowel movements and abdominal wall integrity status remained well. The patient is still under follow-up.

## Discussion

Intestinal LMS is a slow-growing but aggressive malignancy often diagnosed late when curative treatment is ineffective. It is challenging to detect on abdominal computed tomography, with nonspecific symptoms such as vomiting and epigastric discomfort, causing delays [8]. Risk factors include inflammatory bowel diseases, Lynch syndrome, and polyposis syndromes [9]. These tumors commonly affect the retroperitoneum, uterus, vascular wall, and soft tissue, presenting with chronic pain, melena, or anemia [3]. A review of 13 cases [1, 3–5, 8–12] showed a mean age of 69.8 years, a 2:1 male predominance, and a 38.5% mortality rate. The most common presenting symptom was abdominal pain. Tumors were primarily located in the ileum (Table 1).

**Table 1:** Review of 13 cases of LMS of the small intestine

**Authors, year**	**Study design**	**No. of cases**		**Age/Sex**	**Clinical presentation**	**Medical history**	**Surgical history**	**Diagnostic method**	**Immunohistochemistry (+), (−)**					**Tumor site**	**Co-occurrence**	**Treatment**	**Outcome and follow-up**
									**CD117**	**CD34**	**DOG1**	**SMA**	**DS**				
Pilipović-Grubor *et al.,* 2023 [1]	Case report	1		55y/F	Abdominal pain, nausea, vomiting, loss of appetite, and diarrhea	Radiation for endometrial cancer and high ovarian tumor markers	Pelvic surgery for endometrial cancer	Ultrasound, X-ray, CT, MRE, Histo, and IHC	(−)	(−)	(−)	(+)	(+)	Ileum	Mesenteric involvement	Partial bowel resection with ileo-ileal anastomosis	NA
Bouassida *et al.* 2022 [3]	Case report	1		65y/M	Paroxysmal abdominal pain	None	None	Colonoscopy, CT, MRI, Histo, and IHC	(−)	NA	(−)	NA	(+)	Ileum	None	Surgical resection	Alive with no recurrence at 1 year
Zhou *et al,* 2024 [4]	Case report	2	Case 1	70y/M	Abdominal pain and palpable mass	Hypertension and depression	None	CECT, Histo, and IHC	(−)	(−)	(−)	(+)	(+)	Terminal ileum	None	Right hemicolectomy	Died of infectious shock within 9 months
			Case 2	66y/M	Abdominal pain and diarrhea	Invasive lung adenocarcinoma	Radical surgery for lung cancer	CECT, Histo, and IHC	(−)	(−)	(−)	(+)	(+)	Duodenum	None	Segmental duodenal resection	No recurrence after a 7-month follow-up.
Ferrari *et al.* 2020 [5]	Case series	4	Case 1	83y/F	Bowel obstruction and chronic abdominal pain	Arterial hypertension	Cholecystectomy for cholelithiasis	CT, exploratory laparoscopy, Histo, and IHC	(−)	(−)	NA	(+)	(+)	Jejunum	Lymphadenopathy of the mesentery	Jejunal resection and palliative care	Died after a few days
			Case 2	86y/M	Abdominal discomfort and sub-obstruction	NA	None	Ultrasound, CT, percutaneous biopsy, Histo, and IHC	(−)	(−)	NA	(+)	(+)	Ileum	Mesenteric root infiltration and lung metastasis	Trabectedin	Died after 11 months (ischemic stroke)
			Case 3	79y/F	Obstructive mass	NA	Ileal resection	CT, Histo, and IHC	(−)	(−)	NA	(+)	(+)	Ileum	Severe adhesions, colon and rectus muscle infiltration, and postoperative abscess	Ileal resection	Alive with no evidence of recurrence
			Case 4	69y/M	Acute peritonitis and bowel obstruction	Type II diabetes mellitus and chronic kidney disease	Anterior rectal resection for adenocarcinoma	CT, Histo, and IHC	(−)	(−)	NA	(+)	(+)	Ileum	Infiltration of the cecum and abdominal wall	Ileal resection	Alive with no evidence of recurrence at 12 months
Niraj and Richards 2021 [8]	Case report	1		45y/F	Chronic abdominal pain	Gastritis and iron deficiency anemia	None	Endoscopy, CT, and UGN	(−)	NA	NA	(+)	(+)	Small intestine (non-specified site)	None	Ultrasound-guided trigger point injection	Discharged on day 5; high-grade LMS excised.
Kim *et al.* 2020 [9]	Case report	1		80y/M	Abdominal pain, palpable mass	Non-small cell lung cancer	Ileocecal resection	CT, biopsy, Histo, and IHC	(+)*	NA	(−)	(+)	(−)	Ileum	Brain metastasis	Surgical resection	Died after 3 months
Mazzotta *et al.* 2020 [10]	Case report	1		90y/M	Abdominal pain, nausea, and occlusion.	Hypertension and dyslipidemia	Inguinal hernia repair and hemorrhoidectomy	CT, X-ray colonoscopy, and MRI	(−)	(−)	(−)	(+)	(+)	Ileum	Ischemic bowel and mesenteric lymphadenopathy	Ileocecal resection	No complications and no further treatment.
Wilt *et al.* 2024 [11]	Case report	1		53y/M	Abdominal pain, nausea, and vomiting	DVT, gout, and type 2 diabetes	Right nephrectomy	CT, X-ray, Histo, and IHC	(−)	(−)	(−)	(+)	(+)	Terminal ileum	Adherence to the peritoneum, bladder, and sigmoid colon.	Surgical resection	Local recurrence within 8 weeks
Abou El Joud and Abbasi 2021 [12]	Case report	1		67y/M	Abdominal bloating, weight loss, and varicose veins	Untreated hepatitis C	None	Ultrasound, CT, biopsy, Histo, and IHC	(−)	NA	(−)	(+)	(+)	Small bowel (non-specified site)	Lung and liver metastasis and vena cava compression	Palliative care	Died within 2 months

Y, year. M, male. F, female. NA, non-available. DVT, deep vein thrombosis. CT, computed tomography. PET, positron emission tomography. Histo, histology. IHC, immunohistochemistry. MRE, magnetic resonance elastography. MRI, magnetic resonance imaging. CECT, contrast-enhanced computed tomography. SMA, smooth muscle actin. DS, desmin. (−), negative. (+), positive. (+)*, weak positive. UGN, ultrasound-guided needling.

Complete resection with clear margins is the only curative treatment for LMS, but recurrence or metastasis occurs in up to 40% of cases post-excision [11]. Surgical resection is limited to localized cases. In this case, a transverse elliptical incision removed the tumor and 15 cm of normal bowel tissue. Bouassida *et al.* and Zhou *et al.* emphasize clear margins to reduce recurrence risk [3, 4].

Immunohistochemical analyses used were CD117, CD34, DOG1, SMA, and DS. Most cases showed SMA and DS positivity, with CD117 typically negative. CD117 expression was consistently negative except in Kim *et al.*’s report, where there was weak positive staining [9]. A false-positive CD117 result in this case was corrected on repeat staining.

Several reviewed cases highlight the aggressive nature of LMS, with poor survival outcomes. Ferrari *et al.* reported two deaths: one post-jejunal resection in palliative care and another from an ischemic stroke 11 months after trabectedin treatment [5]. Abou El Joud and Abbasi documented a fatal case within 2 months due to metastases causing vena cava compression [12]. Zhou *et al.* noted death from septic shock 9 months post-hemicolectomy [4], while Kim *et al.* reported brain metastasis-related death 3 months post-surgery [9]. In contrast, the present patient had a stable, complication-free recovery at 4 months.

In conclusion, LMS often presents with mild, nonspecific symptoms such as abdominal pain, which may be overlooked until the disease progresses to severe complications. Early medical evaluation, especially in elderly patients, is crucial to rule out serious conditions such as LMS and enable timely intervention.
